# Soil Chromium Accumulation in Industrial Regions across China: Pollution and Health Risk Assessment, Spatial Pattern, and Temporal Trend (2002–2021)

**DOI:** 10.3390/toxics11040363

**Published:** 2023-04-11

**Authors:** Yifan Li, Siyi Pan, Lubin Wang, Fei Jia, Feiyu Lu, Jiyan Shi

**Affiliations:** 1 Department of Environmental Engineering, College of Environmental and Resource Science, Zhejiang University, Hangzhou 310058, China; 2 Zhejiang Jiuhe Geological and Ecological Environment Planning and Design Company, Huzhou 313002, China

**Keywords:** chromium pollution, industrial regions, pollution assessment, health risk assessment, spatial distribution, temporal trend

## Abstract

This study conducted a nationwide specific assessment of soil chromium (Cr) contamination status in 506 of China’s industrial regions. The overall soil Cr concentrations were 0.74–37,967.33 mg/kg, and the soil Cr content in 4.15% of the regions exceeded the reference screening value (2500 mg/kg). Geochemical accumulation index (*I_geo_*) and monomial potential ecological risk index (*E*) revealed Cr salt production and tanning were the primary control industries. The non-carcinogenic risks posed by Cr salt production and tanning industries were higher than the national average values, and children were the most vulnerable groups. The heavily polluted regions were mainly located at the Yangtze River Delta, the Bohai Rim, the Pearl River Delta, the Yangtze River Basin, and the Yellow River Basin. The Yangtze River Delta was further identified as the high priority control area based on the class distribution of *I_geo_* and *E*. Regression analysis showed the soil Cr concentrations in industrial regions increased during 2002–2009 and then turned into a declining trend in 2009–2021. This paper gives detailed insights into soil Cr pollution status in industrial regions across China and the results may serve as references for formulating tailored control measures for different industries and areas.

## 1. Introduction

Chromium (Cr) is one of the most common heavy metal contaminants in soil [1]. As an important chemical material, Cr salt is widely used in electroplating, tanning, pigment, and many other industries, resulting in extensive Cr release into the environment along with industrial wastes [2,3]. In 2014, the Ministry of Ecology and Environment of China and the Ministry of Land and Resources launched the National Survey Bulletin on Soil Pollution Status, which showed the exceeding rate of Cr had reached 1.1% in China [4]. Long-term exposure to Cr may induce Cr ulcers, allergic dermatitis, liver failure, kidney injury, and even cancer [5,6]. Due to the strong toxicity of Cr, the United States Environmental Protection Agency (USEPA) has included Cr in the top contaminants list, and Cr is defined as the fifth potential toxic element according to the Comprehensive Environmental Response, Compensation, and Liability Act [7]. In China, Cr is also listed as one of the key heavy metal pollutants to be monitored as a priority [8].

Soil Cr pollution is widespread in industrial regions in China and preliminary statistics have shown there may be thousands of plots polluted by Cr [9]. In recent years, the Chinese government has been making efforts to control the Cr pollution problems in industrial regions, and emphasis was put on some key industries including mining, smelting, Cr salt production, tanning, electroplating, and so on. Previous research mainly focused on certain site types such as mining or smelting areas [10,11,12], however, soil Cr pollutions widely exist in many other industries. Studies have shown that in some major Cr-related industries such as Cr salt production and tanning industries, the soil Cr concentrations can reach hundreds of times of the background value and cause serious soil Cr pollution [3,13]. At present, the national soil Cr pollution levels of the major Cr-related industries such as the Cr salt production, tanning, and electroplating industries were rarely studied. The previous assessments merely aimed at mining or smelting areas, which may not fully represent the national soil Cr pollution status and may overlook important information for pollution control. Moreover, existing studies mostly only analyzed the soil heavy metal concentrations and assessed their pollution levels and ecological risks [14,15,16]. Common assessment indexes include the Pollution index (*P_i_*), Geochemical accumulation index (*I_geo_*), Enrichment Factor (*EF*), Potential ecological risk index (*E* and *PERI*), and so on [17,18,19]. Among them, *I_geo_* and *E* were the most commonly used indexes for assessing the pollution level and ecological risk of single heavy metals [1,20]. For example, Sun et al. [21] evaluated the soil Cr pollution level in an electroplating site by *I_geo_*. Liu et al. [22] used *I_geo_* and *E* to reveal the pollution levels and ecological risks of Cr, Mn, Co, Ni, Cu, Zn, As, and Pb in soils in Lintong District, Shaanxi. However, these indexes only detect the adverse influences of soil heavy metal pollution on the environment, while the health risk impacts of the contaminated soils were insufficiently evaluated. Therefore, a more comprehensive investigation covering various industries and the assessment of environmental and health impacts of the polluted industrial regions is a priority.

Clarifying the spatial-temporal pattern of the pollutants can provide important insights for formulating pollution control measures [23]. In recent years, the Chinese government has been promoting the identification of key pollution areas to establish tailored control measures based on local conditions. In 2018, the Guideline on Strengthening the Prevention and Control of Pollution in Heavy Metal-Related Industry [24] emphasized that the areas with strong public complaints of environmental pollution associated with Cr-related enterprises needed clarification. In 2022, the Opinions on Further Strengthening the Prevention and Control of Heavy Metal Pollution [25] again stressed the need of delineating key control areas. Additionally, the dynamic changes in pollutant emissions were required to be closely monitored. However, most previous studies were aimed at a single site or small regions [26,27], and national-scale spatial patterns and temporal trends of soil Cr concentrations in industrial regions remain unclear. Therefore, it is essential to track and understand the spatial distribution and temporal change to provide valid information for policymakers.

Hence, this study conducted a comprehensive and detailed investigation of soil Cr pollution status in industrial regions across China. The specific objectives were to: (1) assess the soil Cr pollution level, ecological risks, and health risks in China’s industrial regions; (2) depict the spatial patterns of soil Cr pollution and distinguish priority areas; (3) analyze the temporal variations of soil Cr concentrations in industrial regions; (4) and provide indications for national soil Cr pollution control.

## 2. Materials and Methods

### 2.1. Data Collection

The present work reviewed a large number of papers containing soil Cr concentrations in industrial regions on the Web of Science, China National Knowledge Infrastructure (CNKI), and China WanFang Literature Database. The search terms included “soil pollution”, “Cr”, “China”, “industrial sites”, and so on. The criteria for paper screening were presented in Text S1. After data screening, a database of 506 industrial regions was compiled. The following information was extracted: (1) the title; (2) the first author; (3) the year of publication; (4) the location of the study area; (5) the sampling depth; (6) the sampling year; and (7) the soil Cr concentration. The collected industrial regions and the originating papers were listed in Table S1.

The collected industrial regions involved major Cr-related industries such as Cr salt production, tanning, and electroplating sites, as well as some other typical industry areas with heavy metal pollution such as mining areas. Only one or a few regional data were extracted from each paper to avoid misunderstandings in spatial distribution patterns resulting from centralized sampling. More than 80% of the soil samples were collected within 50 cm of soil depth, which corresponds to the surface layer according to Technical Guidelines for Risk Assessment of Soil Contamination of Land for Construction (HJ 25.3—2019). Therefore, the present work is meant to reflect the soil Cr pollution status in surface soils in China’s industrial regions.

The background value of soil Cr concentration in China and for different provinces was reported by China National Environmental Monitoring Center [28]. Detailed information can be found in Table S2.

### 2.2. Geochemical Accumulation Index (I_geo_)

The *I_geo_*, also called the Muller index, is widely used to evaluate the pollution levels of soil heavy metals [29]. It is calculated by comparing the measured heavy metal contents with the background values. The specific calculation equation of *I_geo_* is as follows:

(1) $$I_{geo}={log}_{2}(\frac{C_{n}}{{1.5B}_{n}})$$

where *C_n_* is the measured concentration of the heavy metal in the soil samples (mg/kg), and *B_n_* is the background value of the heavy metal in the soil (mg/kg). The constant 1.5 is used to eliminate the influence of sedimentary characteristics, rock geology, and other factors on baseline data. According to the value of the *I_geo_*, soil quality is classified into 7 classes [30]. The class divisions and their corresponding pollution levels are listed in Table S3.

### 2.3. Monomial Potential Ecological Risk Index (E)

*E* was used to quantitatively describe the potential ecological risk of a single heavy metal in soils. This method was first proposed by Hakanson [31] and was advantaged as simultaneously considering the concentration of heavy metals and their ecological and environmental effects with toxicology. The *E* of a given metal is defined as:

(2) $$E=T_{n}\times C={\;T}_{n}\times \frac{C_{n}}{B_{n}}$$

where *E* is the monomial potential ecological risk index of a single heavy metal; *T_n_* is the toxic response coefficient of the heavy metal (Cr = 2) [1]; *C* is the contamination factor of the heavy metal; *C_n_* is the measured concentration of the heavy metal in the soil samples (mg/kg), and *B_n_* is the background value of the heavy metal in the soil (mg/kg). The categorization of *E* and the corresponding ecological risk levels are shown in Table S4.

### 2.4. Health Risk Assessment

A health risk assessment model proposed by USEPA was adopted [32]. The population was divided into three groups (i.e., children, adult females, and adult males) according to their different behavioral and physiological performances. Notably, since it was difficult to obtain Cr (VI) concentration for each study area based on literature retrieval, here we only considered the non-carcinogenic risk of Cr. More methodological details of the health risk assessment can be found in Text S2. The toxicological parameters for Cr were provided in Table S5.

### 2.5. Monte Carlo Simulation

The process of health risk assessment involves considerable uncertainty due to an insufficient understanding of the variability in individual human characteristics and environmental systems, as well as the limited availability of the sites’ information [33]. Monte Carlo simulation is one of the most useful tools to quantify uncertainties. The Monte Carlo simulation was conducted using the Oracle Crystal Ball platform (Oracle Corporation, Vallejo, CA, USA). The computation simulation was performed 10,000 times. Detailed information on parameter distribution in Monte Carlo simulation was presented in Table S6.

## 3. Results

### 3.1. Soil Cr Levels in Industrial Regions across China

**Figure 1:**
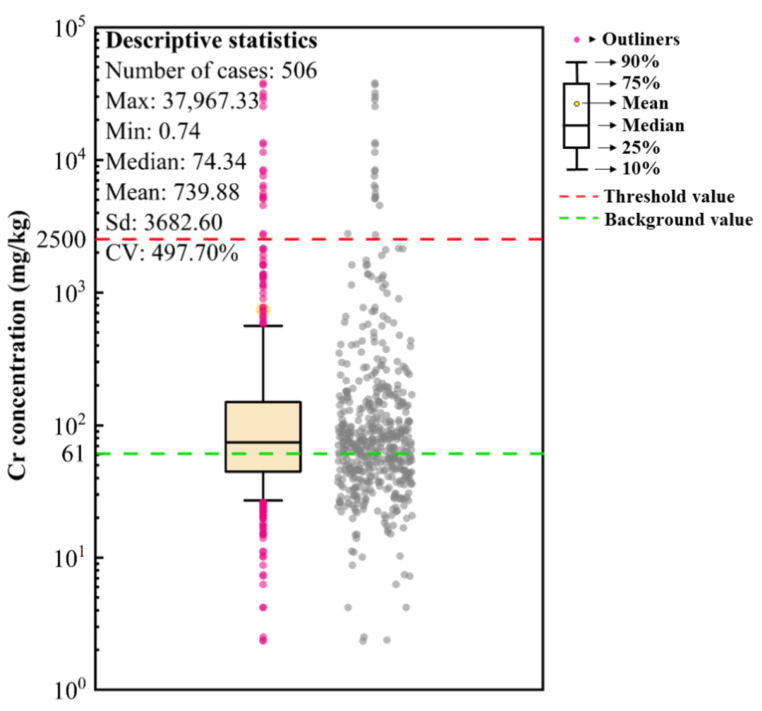
The boxplot and the descriptive statistics of the soil Cr concentrations in industrial regions across China.

The Figure 1 shows the boxplot and the descriptive statistics of the soil Cr concentrations in industrial regions across China. The overall range of soil Cr concentrations was 0.74–37, 967.33 mg/kg, with the mean value being 739.88 mg/kg. The coefficient of variation (CV) reached 497.70%, indicating that soil Cr concentrations in industrial sites were intensely affected by human activities [34]. Compared with the national soil Cr background value (61 mg/kg), 59.68% of the sites’ soil Cr concentrations exceeded the background value. As for the over-standard rate, the screening value for Cr was selected from Beijing standard DB11T811-2011 [35], in which the total Cr threshold value in industrial regions was set as 2500 mg/kg. Compared with this threshold value, the soil Cr over-standard rate in industrial sites was 4.15%.

### 3.2. Pollution and Ecological Risk Assessment

To evaluate the pollution levels and the ecological risks for Cr in industrial regions across China, the *I_geo_* and *E* of each study area were calculated. This study extracted six main region types in the collected data including the mining areas (M), the heavy metal processing and smelting areas (S), the E-waste treatment and disposal sites (E-w), the electroplating sites (El), the tanning and the tannery sludge storage sites (T), and the Cr salt production and chromite ore processing residue disposal areas (C). Their pollution levels and ecological risks were evaluated, respectively.

**Figure 2:**
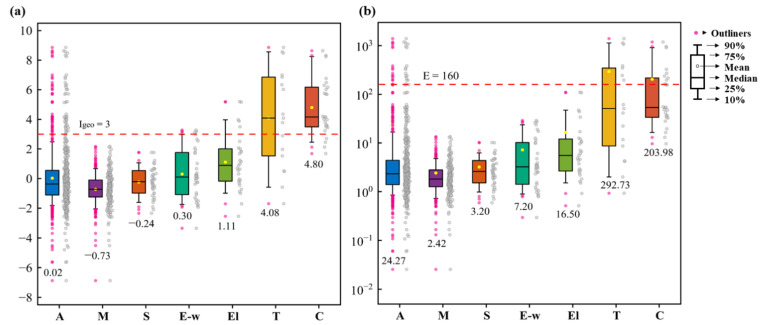
Boxplot of the (**a**) *I_geo_* and (**b**) *E* of all the collected regions and for different region types. (A: all the collected regions; M: mining areas; S: heavy metal processing and smelting areas; E-w: E-waste treatment and disposal sites; El: electroplating sites; T: tanning and the tannery sludge storage sites; C: Cr salt production and chromite ore processing residue disposal areas. The number below each boxplot represents the mean value.).

The average *I_geo_* and *E* values of all the collected regions were 0.02 and 24.27, respectively (Figure 2). The results illustrated that the overall soil Cr pollution in industrial regions in China was mild and the Cr-contaminated soils posed a low ecological risk to the environment. However, an evident disparity in pollution levels was observed between different types of regions. The mean *I_geo_* values for different industrial region types were in the order of C (4.80) > T (4.08) > El (1.11) > E-w (0.30) > S (−0.24) > M (−0.73) (Figure 2a). The *E* values showed a similar order of T (292.73) > C (203.98) > El (16.50) > E-w (7.20) > S (3.20) > M (2.42) (Figure 2b). As observed from the average *I_geo_* values, the Cr salt production and the tanning industries were both rated as heavily contaminated by Cr. Likely, the average *E* values suggested the Cr-contaminated soils in these two industries posed high ecological risks to the environment. Both evaluations indicated the Cr salt production and tanning industries suffered the most serious soil Cr pollution problem.

**Figure 3:**
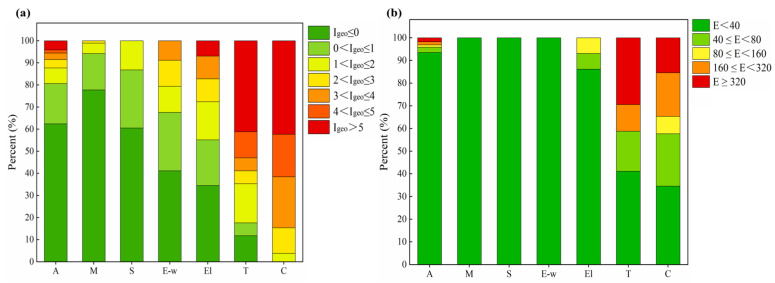
Class distribution of the (**a**) *I_geo_* and (**b**) *E* of all the collected regions and for different region types. (A: all the collected regions; M: mining areas; S: heavy metal processing and smelting areas; E-w: E-waste treatment and disposal sites; El: electroplating sites; T: tanning and the tannery sludge storage sites; C: Cr salt production and chromite ore processing residue disposal areas.).

As for the class distributions of the *I_geo_* and *E*, it is observed that the *I_geo_* in 42.31% of the Cr salt production sites and 41.18% of the tanning sites exceeded 5, which means these sites were extremely polluted by Cr (Figure 3a). Additionally, the class distribution results of *E* showed that 15.38% of the Cr salt production sites and 29.41% of the tanning sites posed significant high risks to the environment (Figure 3b). Therefore, the Cr salt production and the tanning industries should be primarily controlled for soil Cr pollution prevention.

### 3.3. Health Risk Assessment

**Figure 4:**
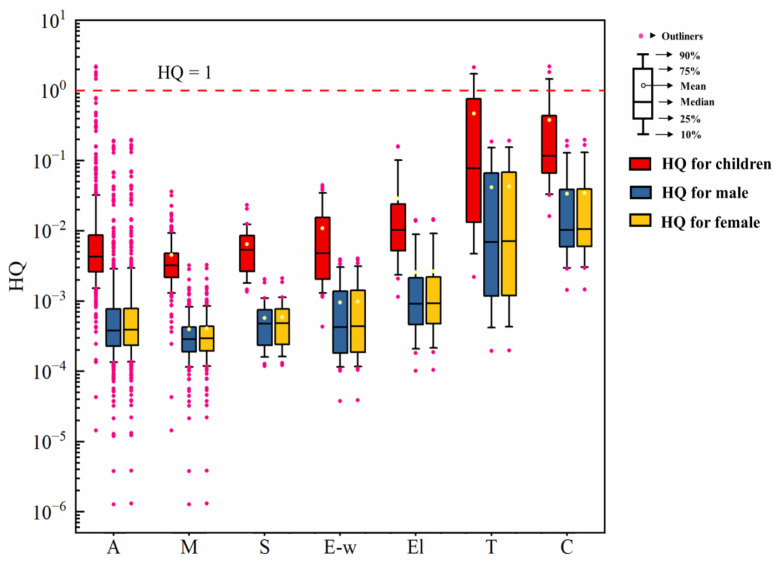
The non-carcinogenic risk of all the collected regions and for different region types. (A: all the collected regions; M: mining areas; S: heavy metal processing and smelting areas; E-w: E-waste treatment and disposal sites; El: electroplating sites; T: tanning and the tannery sludge storage sites; C: Cr salt production and chromite ore processing residue disposal areas.).

The non-carcinogenic risks of all the collected regions and for different industrial region types are shown in Figure 4. In most of the collected regions, the HQ values were below 1, representing the national non-carcinogenic risks level of soil Cr was acceptable. However, for some cases in the Cr salt production and tanning industries, the HQ values were beyond 1 for children. Table S7 shows the statistical information of the non-carcinogenic risk of all the collected regions and for different region types. The national average HQ values of Cr for children, males, and females were 4.28 × 10^−2^, 3.78 × 10^−3^, and 3.88 × 10^−3^, respectively. Children were exposed to the highest health risks. The average HQ values for children in the Cr salt production and the tanning industries were 3.82 × 10^−1^ and 4.73 × 10^−1^, equaling 8.92 and 11.05 times of the national mean HQ value for children. The proportions of HQ surpassing 1 in the Cr salt production and tanning industries were 3.85% and 5.88%. Therefore, the health risks introduced by soil Cr pollution in the Cr salt production and tanning industries should be paid special attention to and children should be chiefly protected.

### 3.4. Spatial Distribution Pattern

**Figure 5:**
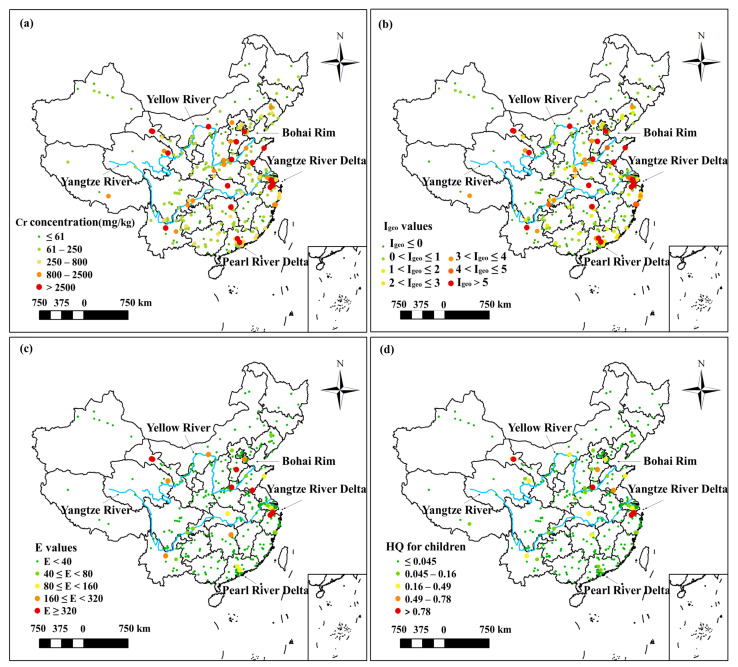
Spatial distribution maps of the (**a**) soil Cr concentrations; (**b**) *I_geo_* values; (**c**) *E* values; and (**d**) HQ values for children.

The spatial distribution maps of the soil Cr concentrations, *I_geo_* values, *E* values, and HQ values for children of the collected regions were illustrated in Figure 5. Observed from the soil Cr concentration and pollution level maps (Figure 5a,b), the heavily polluted industrial regions were primarily located around five areas, which are the Yangtze River Delta, the Bohai Rim, and the Pearl River Delta, the Yangtze River Basin, and the Yellow River Basin. Among these areas, the industrial regions were most intensively distributed in the Yangtze River Delta. As for the distribution of industrial regions with high ecological and health risks, the Yangtze River Delta and the Bohai Rim were more concerning areas (Figure 5c,d), whereas the ecological and health risks posed by Cr-contaminated soils in the other three areas are lower.

**Figure 6:**
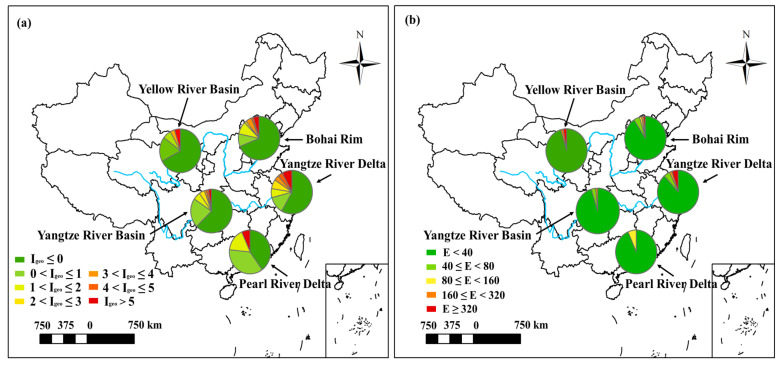
Class distribution of the (**a**) *I_geo_* and (**b**) *E* of the Yangtze River Delta, the Bohai Rim, the Pearl River Delta, the Yangtze River Basin, and the Yellow River Basin.

To further compare the pollution status among different areas, the class distribution of *I_geo_* values and *E* values were shown in Figure 6. For the class distribution of the *I_geo_*, it was observed that the Yangtze River Delta had the highest proportion of the “extremely contaminated” regions with the number of 7.77%, followed by 6.67% for the Pearl River Delta and 4.68% for the Bohai Rim. As for the class distribution of the *E* values, the proportion of the regions with “significant high ecological risks” in the Yangtze River Delta still outranked other areas, reaching 4.85%. Additionally, among all the 21 regions with soil Cr concentration exceeding 2500 mg/kg, 8 were located at the Yangtze River Delta, accounting for 38.1% of the totality (Table S8). Thus, the Yangtze River Delta is the high-priority area for soil Cr pollution control.

### 3.5. Temporal Trend of Soil Cr Concentrations in Industrial Regions

**Figure 7:**
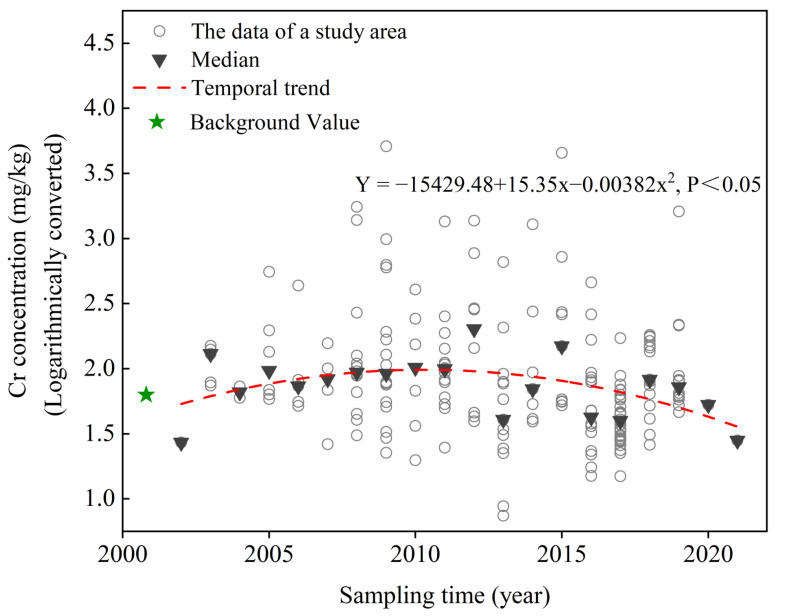
Temporal trend of soil Cr concentrations in industrial regions during 2002–2021.

A regression analysis was carried out using the median Cr concentration of each year to extrapolate the temporal trend during 2002–2021 as previous studies had done [23,36]. The origin data of soil Cr concentrations showed a strong positive skewed distribution, which is unsuitable for subsequent statistical analysis. Therefore, a logarithmic transformation was applied before further analysis. It can be observed in Figure 7 that the soil Cr concentrations in industrial regions showed a growing trend initially. However, the growth rate kept slowing down. The curve turned into a declining trend around 2009, indicating the input-output balance of soil Cr had reversed. Afterward, the soil Cr concentration in industrial regions kept the decreasing trend during 2009–2021.

## 4. Discussion

### 4.1. Priority Control Industries and Comparison with Other Studies

This study conducted a comprehensive assessment of soil Cr pollution status in industrial regions across China based on 506 regions’ data. Compared with existing studies, this study not only assessed the national pollution status, but also specifically quantified the soil Cr accumulation level, ecological risks, and health risks for six different industrial region types. The results showed the Cr salt production and the tanning industries were most polluted by Cr, followed by the electroplating industries and the E-waste treatment and disposal sites, whereas the soil Cr pollution in the smelting and the mining areas were light.

The pollution assessment results for different industrial region types in this study are consistent with many previous studies focusing on a single industry. For example, Liu et al. [10] identified a low soil Cr pollution level (average *I_geo_* = −0.7) and low ecological risk (*E* < 40) in their research of a national assessment of heavy metal pollution status in mining-affected areas. Similar results were also acquired by Li et al. [37], who found the average *I_geo_* value for Cr was −0.56 in China’s mine soils. Li et al. [11] and Yang et al. [38] conducted a soil heavy metal pollution evaluation in smelting regions in China and they both found few smelting regions with exceeding soil Cr concentrations. He et al. [39] reviewed the heavy metal pollution situation of e-waste recycling regions and found most e-waste recycling regions were with low soil Cr pollution.

As few studies have quantified the national soil Cr pollution levels, ecological risks, and health risks of the Cr salt production and tanning industries, here we compare the concentration range of our results with previous reviews. Meng [40] reported a concentration range of 23.5–56,000 mg/kg in chromite ore processing residue disposal areas in China, and the soil Cr concentration range in tanning regions was reported to range from 33.4 to 59,400 mg/kg by Xu et al. [41]. The soil Cr concentration in Cr salt production and tanning industrial regions identified in this study was 279.7–37,967.33 mg/kg and 38–36,871 mg/kg, respectively. It can be seen that the Cr concentration ranges in our results are consistent with previous reviews and the smaller ranges are mainly due to the exclusion of some documents that do not meet the screening requirements of this article. Therefore, the evaluation results of the pollution situation in different industries in this study are relatively reliable and the government should keep a close watch on the Cr salt production and the tanning industries for soil Cr pollution control.

### 4.2. Priority Control Areas and Potential Future Focus

The present study revealed some hotspots for soil Cr pollution such as the Yangtze River Delta, the Bohai Rim, the Pearl River Delta, the Yangtze River Basin, and the Yellow River Basin. The Yangtze River Delta, the Bohai Rim, and the Pearl River Delta have long been China’s important industrial zones since the reform and opening up and the intensive industrial activities have caused soil heavy metal pollution in these areas [42,43]. These areas are often related to thriving Cr-related industries. For example, Haining in Jiaxing, Zhejiang is the largest leather production base in China [13], and Xinji in Hebei province is one of China’s most important leather-producing areas [26]. Therefore, the discharge of industrial wastes from Cr-related industries may lead to prominent soil Cr pollution issues in these areas. As for the Yangtze River Basin and the Yellow River Basin, these two areas are major parts of the Yangtze River Economic Belt and the Yellow River Economic Zone [44,45]. The two economic zones have been through sharp industry developments in recent years and Hu et al. [46] identified a gathering tendency of chemical manufacturing industries in middle China around the Yangtze River Basin and the Yellow River Basin during 2008–2014. Therefore, the industrial activities of some Cr-related chemical enterprises may contribute to the soil Cr pollution in these areas.

Compared with previous studies, the hotspots have also been confirmed as abundant of industrial legacies with severe heavy metal pollution by Peng et al. [47]. Likely, Li et al. [48] explored the spatial pattern of heavy metal pollution risks of industrial cultivated lands across China and found the high-risk regions were mostly distributed in the Yangtze River Delta, the Pearl River Delta, and some other provinces such as Tianjin, Hebei, Sichuan, Chongqing, Hunan, and Henan, which are exactly located at the Bohai Rim, the Yangtze River Basin and the Yellow River Basin. Additionally, compared with the data provided by Wang et al. [9] (Figure S1), the spatial pattern identified in the present study well reflected the main distribution areas of Cr-related industries in China. Thus, the spatial distribution patterns identified in the present study are proven to be trustworthy.

After identifying the priority control areas, this study further compared the pollution and ecological risk levels in the five hotspots. Assisted by the results of class distributions of *I_geo_* and *E*, the Yangtze River Delta was acknowledged as the most polluted area for Cr and should be controlled as a high priority. Previous studies have also reported that, compared with the Pearl River Delta and the Jing-Jin-Ji district, the industrial activities presented a more significant impact on the heavy metal accumulation in the Yangtze River Delta [49].

It is worth noting that, as the soil heavy metal pollution issue in the Yangtze River Delta arises growing attention, the government has now taken actions to promote pollution-intensive industries to transfer away from the Yangtze River Delta and thus releasing the environmental burdens in this area [50]. With the proposal of constructing the Yangtze River Economic Belt and the Yellow River Economic Zone, these two areas are undergoing rapid economic development and many pollution-intensive industries are relocating along the Yangtze River Basin and the Yellow River Basin [51,52]. However, it has been found that the industry transfer could render the recipient areas vulnerable to extra environmental pollution [53]. Therefore, the Yangtze River Basin and the Yellow River Basin may become a future focus for pollution prevention. Tightened emission limits and sounder supervision policies may be needed to avoid possible pollution aggravation in these areas.

### 4.3. Interpretations of the Temporal Trend

According to the temporal trend analysis, the soil Cr concentrations in industrial regions in China initially increased during 2002–2009 and then turned into a declining trend in 2009–2021. Similarly, Li et al. [36] analyzed the temporal variations of soil Cr concentration in agricultural soils in China and found the soil Cr accumulation first increased during 1989–2010 and then slowly decreased during 2011–2016. Huang et al. [54] found the accumulation of Cr in agricultural soils in China had decreased since 2012. For the interpretations of the temporal trend, previous studies have largely credited the rapid industry developments in China for the increasing pollutant concentrations in the environment [19,55]. Contrarily, the formulations and implementations of the control measures may favor the alleviation of soil pollution [56,57].

Tracing the development history of Cr-related industries, the growing soil Cr concentrations during 2002–2009 may be related to insufficient supervision during Cr-related industries’ development. For example, in the past, many chromium ore processed residues generated in the Cr salt production industry were simply stacked on the ground without any cover or impervious barriers [58,59]. Similarly, tannery sludges produced during tanning processes were often simply landfilled without pretreatment to fix or remove the toxic elements [60]. Cr in these wastes could easily migrate into the soil and cause serious pollution. With the constant industrial emissions to the environment, the soil Cr concentrations appeared to increase in 2002–2009. Fortunately, the Chinese government quickly noticed the pollution issues and has taken many measures to control the soil Cr pollution such as promoting cleaner production, tightening emission restrictions, and closing pollution-intensive enterprises. For example, in 2009, the Ministry of Industry and Information Technology of China launched the Guideline on Restructuring of Leather Industry [61], which encouraged tannery enterprises to adopt new, cleaner crafts. In 2016, the Action Plan on Soil Pollution Prevention and Control addressed the supervision of pollutant emissions of heavy-metal-related enterprises, and those substandard ones should be shut down [8]. Therefore, as more and more regulations are put forward, the soil Cr input has been effectively restrained. Moreover, the promotion of remediation projects in Cr-polluted sites further strengthen the output of soil Cr and alleviate the pollution [62]. Preliminary statistics showed more than 10 remediation projects for Cr-polluted sites had been carried out by the end of 2019 [9] and more policies have been launched for regulating the remediation processes of the contaminated sites [63]. Therefore, under the joint effects of the control measures and the promotion of remediation, the soil Cr accumulation in industrial regions decreased during 2009–2021.

However, it should be noted that although the Chinese government has passed numerous policies to control soil pollution, the implementation of some was poorly performed [64]. Therefore, despite the positive effects of the emission control measures and the remediation of contaminated sites, it is important to monitor the proceedings of the policy implementation and obtain timely feedback to fully address the soil Cr pollution problems.

### 4.4. Comparison with Existing Studies

The present work made some progress compared with existing studies. First of all, the data size of the collected regions in the present study is larger. For example, Peng et al. [47] compiled a database of industrial legacies in China and documented the heavy metal concentrations in 185 legacies. Yang [65] et al. evaluated the soil heavy metal health risks in 402 industrial regions. The present work included 506 industrial regions, and the larger size of the collected data may enhance the representativeness of the results. Moreover, this study is specifically aimed at industrial regions. Previous studies may simultaneously assess the pollution status in industrial regions and agricultural soils and the calculations were mixed [1,66]. However, due to the high heterogeneity of the soil environment, the soil heavy metal pollution characteristics may differ a lot in different land-use types [67]. Hence, the mixed calculations may enlarge the uncertainty of the data and confuse the identification of priority areas. Therefore, this study can provide more targeted guidance for formulating control measures in industrial regions without interference from other land-use types. Finally, this study included more industrial region types. Previous studies mostly focus on only mining or smelting areas [10,11,12], whereas major Cr-related industries such as the Cr salt production, tanning, and electroplating industries are less studied. Therefore, this study provides a more comprehensive and deeper understanding of soil Cr pollution status in industrial regions in China and may help policymakers to make efficient control measures.

### 4.5. Limitations

Firstly, due to the limited availability of information on industrial regions, the number of collected regions in this study is still small compared to the real number in China. Secondly, publication bias may exist as researchers tend to pay more attention to the heavily polluted enterprises, and overestimation of the soil Cr pollution level may be introduced accordingly. Last, as most studies did not disclose the Cr (VI) concentrations in soils, the carcinogenic health risk of Cr (VI) is not considered yet.

## 5. Conclusions

This study comprehensively evaluated the soil Cr pollution status in industrial regions across China based on data from 506 regions. The specific soil Cr accumulation level, ecological risks, and health risks were quantified. Through the statistical analysis, the key industries and areas for soil Cr pollution control were identified and the temporal variation of soil Cr concentrations was analyzed.

The results showed that the soil Cr concentrations ranged from 0.74 to 37,967.33 mg/kg in industrial regions in China. The soil Cr concentrations were largely affected by anthropogenic activities. In 4.15% of the collected regions, the soil Cr concentrations exceeded the corresponding screening value (2500 mg/kg). The national soil Cr pollution was mild and the corresponding ecological risk in industrial regions was low according to the national average *I_geo_* and *E* values. However, different industries exhibited varied pollution levels. The Cr salt production and tanning industries were heavily polluted by Cr and the soil Cr pollution posed high ecological risks to the environment. Health risk assessment showed that the soil Cr pollution in the Cr salt production and tanning industries imposed much higher health risks compared to the national mean values and children were at the highest risk. In summary, the Cr salt production and tanning industries should be primarily controlled. For the spatial distribution pattern, some pollution hotspots such as the Yangtze River, the Bohai Rim, the Pearl River, the Yangtze River Basin, and the Yellow River Basin were revealed. Among the hotspots, the Yangtze River Delta area was at the highest pollution level and ecological risk and thus should be controlled as a high priority. The temporal analysis showed the soil Cr concentrations in industrial regions followed an increasing trend during 2002–2009 and turned into a downward trend during 2009–2021. This paper provides informative indications on soil Cr pollution control, and the results may help establish differentiated control policies for key industries and areas in the future.

## Supplementary Materials

The following supporting information can be downloaded at: https://www.mdpi.com/article/10.3390/toxics11040363/s1, Table S1: Information of each study area and its originating literature; Table S2: Background values of soil Cr concentration in China and for different provinces (mg/kg); Table S3: Classification of the geochemical accumulation index (*I_geo_*); Table S4: Classification of the monomial potential ecological risk index (*E*); Table S5: Toxicological parameters of Cr; Table S6: Parameter distribution in Monte Carlo simulation; Table S7: Statistical information of the non-carcinogenic risk of all the collected regions and for different region types; Table S8: Industrial regions with soil Cr concentration exceeding the screening value (2500 mg/kg); Text S1: The criteria for paper screening; Text S2: The methodological details for health risk assessment; Figure S1: Distribution of major Cr-related industries in China.

## References

[1] Hu B., Shao S., Ni H., Fu Z., Shi Z. (2020). Current Status, Spatial Features, Health Risks, and Potential Driving Factors of Soil Heavy Metal Pollution in China at Province Level. Environ. Pollut..

[2] Pradhan D., Sukla L.B., Sawyer M., Rahman P.K.S.M. (2017). Recent Bioreduction of Hexavalent Chromium in Wastewater Treatment: A Review. J. Ind. Eng. Chem..

[3] Wang X., Li L., Yan X., Meng X., Chen Y. (2020). Processes of Chromium (VI) Migration and Transformation in Chromate Production Site: A Case Study from the Middle of China. Chemosphere.

[4] Ministry of Ecology and Environment of China, (MEE of China) (2014). National Soil Pollution Survey Bulletin.

[5] Malaviya P., Singh A. (2011). Physicochemical Technologies for Remediation of Chromium-Containing Waters and Wastewaters. Crit. Rev. Environ. Sci. Technol..

[6] Hu B., Jia X., Hu J., Xu D., Xia F., Li Y. (2017). Assessment of Heavy Metal Pollution and Health Risks in the Soil-Plant-Human System in the Yangtze River Delta, China. Int. J. Environ. Res. Public Health.

[7] Xia S., Song Z., Jeyakumar P., Shaheen S.M., Rinklebe J., Ok Y.S., Bolan N., Wang H. (2019). A Critical Review on Bioremediation Technologies for Cr(VI)-Contaminated Soils and Wastewater. Crit. Rev. Environ. Sci. Technol..

[8] State Council of China (2016). The Action Plan for Soil Pollution Prevention and Control. http://www.gov.cn/xinwen/2016-05/31/content_5078467.htm.

[9] Wang X., Li L., Yan X., Tian Y. (2020). Progress in Remediation of Chromium-contaminated Sites. Environ. Eng..

[10] Liu H., Qu M., Chen J., Xu G., Zhang J., Liu M., Kang J., Zhao Y., Huang B. (2022). Heavy Metal Accumulation in the Surrounding Areas Affected by Mining in China: Spatial Distribution Patterns, Risk Assessment, and Influencing Factors. Sci. Total Environ..

[11] Li Q., He L., Wang Y., Cao Y., Gao C., Liu X. (2021). The Characteristics and Distribution of Soil Pollution in Smelting Industry Sites in China. Ecol. Environ. Sci..

[12] Kan X., Dong Y., Feng L., Zhou M., Hou H. (2021). Contamination and Health Risk Assessment of Heavy Metals in China’s Lead–Zinc Mine Tailings: A Meta–Analysis. Chemosphere.

[13] Yuan Y., Yu S., Bañuelos G.S., He Y. (2016). Accumulation of Cr, Cd, Pb, Cu, and Zn by Plants in Tanning Sludge Storage Sites: Opportunities for Contamination Bioindication and Phytoremediation. Environ. Sci. Pollut. Res..

[14] Li S., Wu J., Huo Y., Zhao X., Xue L. (2021). Profiling Multiple Heavy Metal Contamination and Bacterial Communities surrounding an Iron Tailing Pond in Northwest China. Sci. Total Environ..

[15] Fang X., Peng B., Wang X., Song Z., Zhou D., Wang Q., Qin Z., Tan C. (2019). Distribution, Contamination and Source Identification of Heavy Metals in Bed Sediments from the Lower Reaches of the Xiangjiang River in Hunan Province, China. Sci. Total Environ..

[16] Jia Y., Zhang W., Liu M., Peng Y., Hao C. (2022). Spatial Distribution, Pollution Characteristics and Source of Heavy Metals in Farmland Soils around Antimony Mine Area, Hunan Province. Pol. J. Environ. Stud..

[17] Zhang Q., Han G., Liu M., Liang T. (2019). Spatial Distribution and Controlling Factors of Heavy Metals in Soils from Puding Karst Critical Zone Observatory, Southwest China. Environ. Earth Sci..

[18] Khan S., Naushad M., Lima E.C., Zhang S., Shaheen S.M., Rinklebe J. (2021). Global Soil Pollution by Toxic Elements: Current Status and Future Perspectives on the Risk Assessment and Remediation Strategies—A Review. J. Hazard. Mater..

[19] Zhang X., Zha T., Guo X., Meng G., Zhou J. (2018). Spatial Distribution of Metal Pollution of Soils of Chinese Provincial Capital Cities. Sci. Total Environ..

[20] Sun J., Zhao M., Cai B., Song X., Tang R., Huang X., Huang H., Huang J., Fan Z. (2022). Risk Assessment and Driving Factors of Trace Metal(Loid)s in Soils of China. Environ. Pollut..

[21] Sun J., Luo Y., Ye J., Li C., Shi J. (2022). Chromium Distribution, Leachability and Speciation in a Chrome Plating Site. Processes.

[22] Liu J., Kang H., Tao W., Li H., He D., Ma L., Tang H., Wu S., Yang K., Li X. (2023). A Spatial Distribution—Principal Component Analysis (SD-PCA) Model to Assess Pollution of Heavy Metals in Soil. Sci. Total Environ..

[23] Gong Y., Qu Y., Yang S., Tao S., Shi T., Liu Q., Chen Y., Wu Y., Ma J. (2020). Status of Arsenic Accumulation in Agricultural Soils across China (1985–2016). Environ. Res..

[24] Ministry of Ecology and Environment of China (MEE of China) (2018). The Guideline on Strengthening the Prevention and Control of Pollution in Heavy Metal-Related Industry.

[25] Ministry of Ecology and Environment of China (MEE of China) (2022). The Opinions on Further Strengthening the Prevention and Control of Heavy Metal Pollution.

[26] Kong X., Li C., Wang P., Huang G., Li Z., Han Z. (2019). Soil Pollution Characteristics and Microbial Responses in a Vertical Profile with Long-Term Tannery Sludge Contamination in Hebei, China. Int. J. Environ. Res. Public Health.

[27] Li C., Sanchez G., Wu Z., Cheng J., Zhang S., Wang Q., Li F., Sun G., Meentemeyer R. (2020). Spatiotemporal Patterns and Drivers of Soil Contamination with Heavy Metals during an Intensive Urbanization Period (1989–2018) in Southern China. Environ. Pollut..

[28] China National Environmental Monitoring Center (CNEMC) (1990). The Background Concentrations of Soil Elements of China.

[29] Loska K., Wiechuła D., Korus I. (2004). Metal Contamination of Farming Soils Affected by Industry. Environ. Int..

[30] Förstner U., Ahlf W., Calmano W., Kersten M. (1990). Sediment Criteria Development. Sediments and Environmental Geochemistry.

[31] Hakanson L. (1980). An ecological risk index for aquatic pollution control. A sedimentological approach. Water Res..

[32] United States Environmental Protection Agency (USEPA) (1989). Risk Assessment Guidance for Superfund Volume I. Human Health Evaluation Manual (Part A).

[33] Mari M., Nadal M., Schuhmacher M., Domingo J. (2009). Exposure to Heavy Metals and PCDD/Fs by the Population Living in the Vicinity of a Hazardous Waste Landfill in Catalonia, Spain: Health Risk Assessment. Environ. Int..

[34] Wang Y., Duan X., Wang L. (2020). Spatial Distribution and Source Analysis of Heavy Metals in Soils Influenced by Industrial Enterprise Distribution: Case Study in Jiangsu Province. Sci. Total Environ..

[35] (2011). Screening Levels for Soil Environmental Risk Assessment of Sites.

[36] Li X., Zhang J., Ma J., Liu Q., Shi T., Gong Y., Yang S., Wu Y. (2020). Status of Chromium Accumulation in Agricultural Soils across China (1989–2016). Chemosphere.

[37] Li Z., Ma Z., van der Kuijp T.J., Yuan Z., Huang L. (2014). A Review of Soil Heavy Metal Pollution from Mines in China: Pollution and Health Risk Assessment. Sci. Total Environ..

[38] Yang L., Li Y., Liu J., Liang Y., Zhang X. (2022). Current Status and Risk Assessment of Heavy Metal Pollution in China’s Smelters and Surrounding Soils. J. Earth Environ..

[39] He K., Sun Z., Hu Y., Zeng X., Yu Z., Cheng H. (2017). Comparison of Soil Heavy Metal Pollution Caused by E-Waste Recycling Activities and Traditional Industrial Operations. Environ. Sci. Pollut. Res..

[40] Meng F. (2016). Pollution Characteristics of Soils Polluted by Chromium Slag in China. Environ. Pollut. Control..

[41] Xu T., Nan F., Jiang X., Tang Y., Zhang W., Shi B. (2020). Progresses in Research on Sources and Characteristics of Chromium Pollution in Soils and Groundwater of Tannery Sites. Acta Pedol. Sin..

[42] Zhao X., Zhang Q., He G., Zhang L., Lu Y. (2021). Delineating Pollution Threat Intensity from Onshore Industries to Coastal Wetlands in the Bohai Rim, the Yangtze River Delta, and the Pearl River Delta, China. J. Clean. Prod..

[43] Duan Q., Lee J., Liu Y., Chen H., Hu H. (2016). Distribution of Heavy Metal Pollution in Surface Soil Samples in China: A Graphical Review. Bull. Environ. Contam. Toxicol..

[44] Xu X., Yang G., Tan Y., Liu J., Hu H. (2018). Ecosystem Services Trade-Offs and Determinants in China’s Yangtze River Economic Belt from 2000 to 2015. Sci. Total Environ..

[45] Ji Y., Zhang L. (2023). Comparative Analysis of Spatial–Temporal Differences in Sustainable Development between the Yangtze River Economic Belt and the Yellow River Economic Belt. Environ. Dev. Sustain..

[46] Hu Z., Miao C. (2018). The Spatiotemporal Pattern of China’s Pollution Industry Transfer and Its Relationship with Pollution Transfer. Soft Sci..

[47] Peng J., Zhang S., Han Y., Bate B., Ke H., Chen Y. (2022). Soil Heavy Metal Pollution of Industrial Legacies in China and Health Risk Assessment. Sci. Total Environ..

[48] Li K., Wang J., Zhang Y. (2022). Heavy Metal Pollution Risk of Cultivated Land from Industrial Production in China: Spatial Pattern and Its Enlightenment. Sci. Total Environ..

[49] Zhou X.-Y., Wang X.-R. (2019). Impact of Industrial Activities on Heavy Metal Contamination in Soils in Three Major Urban Agglomerations of China. J. Clean. Prod..

[50] Hu J., Liu Y., Fang J., Jing Y., Liu Y., Liu Y. (2019). Characterizing Pollution-intensive Industry Transfers in China from 2007 to 2016 Using Land Use Data. J. Clean. Prod..

[51] Song C., Yin G., Lu Z., Chen Y. (2022). Industrial Ecological Efficiency of Cities in the Yellow River Basin in the Background of China’s Economic Transformation: Spatial-Temporal Characteristics and Influencing Factors. Environ. Sci. Pollut. Res..

[52] Chen J.-X., Zhang Y., Zheng S. (2019). Ecoefficiency, Environmental Regulation Opportunity Costs, and Interregional Industrial Transfers: Evidence from the Yangtze River Economic Belt in China. J. Clean. Prod..

[53] Wei D., Liu Y., Zhang N. (2019). Does Industry Upgrade Transfer Pollution: Evidence from a Natural Experiment of Guangdong Province in China. J. Clean. Prod..

[54] Huang Y., Wang L., Wang W., Li T., He Z., Yang X. (2019). Current Status of Agricultural Soil Pollution by Heavy Metals in China: A Meta-Analysis. Sci. Total Environ..

[55] Huang Y., Deng M., Li T., Japenga J., Chen Q., Yang X., He Z. (2017). Anthropogenic Mercury Emissions from 1980 to 2012 in China. Environ. Pollut..

[56] Yang Z., Li X., Wang Y., Chang J., Liu X. (2021). Trace Element Contamination in Urban Topsoil in China during 2000–2009 and 2010–2019: Pollution Assessment and Spatiotemporal Analysis. Sci. Total Environ..

[57] He M., Shen H., Li Z., Wang L., Wang F., Zhao K., Liu X., Wendroth O., Xu J. (2019). Ten-Year Regional Monitoring of Soil-Rice Grain Contamination by Heavy Metals with Implications for Target Remediation and Food Safety. Environ. Pollut..

[58] Zhang S., Hao X., Tang J., Hu J., Deng Y., Xu M., Zhu P., Tao J., Liang Y., Yin H. (2020). Assessing Chromium Contamination in Red Soil: Monitoring the Migration of Fractions and the Change of Related Microorganisms. Int. J. Environ. Res. Public Health.

[59] Huang S., Peng B., Yang Z., Chai L., Xu Y., Su C. (2009). Spatial Distribution of Chromium in Soils Contaminated by Chromium-Containing Slag. Trans. Nonferrous Met. Soc. China.

[60] Kong X., Wang Y., Ma L., Huang G., Zhang Z., Han Z. (2020). Leaching Behaviors of Chromium(III) and Ammonium-Nitrogen from a Tannery Sludge in North China: Comparison of Batch and Column Investigations. Int. J. Environ. Res. Public Health.

[61] Ministry of Industry and Information Technology of China (2009). The Guideline on Restructuring of Leather Industry. https://www.miit.gov.cn/xwdt/gxdt/ldhd/art/2020/art_eebca094c4364890b28d0dee92af4e58.html.

[62] Wu Y., Li X., Yu L., Wang T., Wang J., Liu T. (2022). Review of Soil Heavy Metal Pollution in China: Spatial Distribution, Primary Sources, and Remediation Alternatives. Resour. Conserv. Recycl..

[63] Li X., Jiao W., Xiao R., Chen W., Liu W. (2017). Contaminated Sites in China: Countermeasures of Provincial Governments. J. Clean. Prod..

[64] Zheng H., Li R., Cao S. (2011). Comment on “Chromium Contamination Accident in China: Viewing Environment Policy of China”. Environ. Sci. Technol..

[65] Yang Q., Li Z., Lu X., Duan Q., Huang L., Bi J. (2018). A Review of Soil Heavy Metal Pollution from Industrial and Agricultural Regions in China: Pollution and Risk Assessment. Sci. Total Environ..

[66] Hu B., Shao S., Ni H., Fu Z., Huang M., Chen Q., Shi Z. (2021). Assessment of Potentially Toxic Element Pollution in Soils and Related Health Risks in 271 Cities across China. Environ. Pollut..

[67] Lee C.S., Li X., Shi W., Cheung S.C., Thornton I. (2006). Metal Contamination in Urban, Suburban, and Country Park Soils of Hong Kong: A Study Based on GIS and Multivariate Statistics. Sci. Total Environ..

