# Supplementary material for: Soil Chromium Accumulation in Industrial Regions across China: Pollution and Health Risk Assessment, Spatial Pattern, and Temporal Trend (2002–2021)
Source: Toxics. 2023 Apr 11;11(4):363. doi: 10.3390/toxics11040363 (PMC10143473; doi:10.3390/toxics11040363)
Supplement: Supplementary file 1 [file toxics-11-00363-s001.zip › Supplementary Material-Texts and Figure.pdf]

# Soil Chromium Accumulation in Industrial Regions across China: Pollution and Health Risk Assessment, Spatial Pattern, and Temporal Trend (2002–2021)

**Yifan Li [1], Siyi Pan [1], Lubin Wang [1], Fei Jia [2], Feiyu Lu [1] and Jiyan Shi [1,*]**

[1] Department of Environmental Engineering, College of Environmental and Resource Science, Zhejiang University, Hangzhou 310058, China
[2] Zhejiang Jiuhe Geological and Ecological Environment Planning and Design Company, Huzhou 313002, China
* Correspondence: shijiyan@zju.edu.cn; Tel.: +86-571-8898-2019

## Supplementary Materials:

### Texts

Text S1: The criteria for paper screening. Page 1-2

Text S2: The methodological details for health risk assessment. Page 2-3

### Figures

Figure S1: Distribution of major Cr-related industries in China. Page 4

**Text S1:** The criteria for paper screening.

To ensure the reliability of the data, the following criteria were used to screen the qualified papers: (1) The location of the study area (accurate to the city at least) should be noted; (2) The chemical analysis methods of the soil samples should be executed according to relevant standards; (3) The mean soil Cr concentration of the region was accessible (e.g., provided in a table, can be extracted in figures with exact coordinates,

or can be calculated with the provided information). (4) The samples should not be road dust, river sediments, or agricultural soils around industrial regions.

**Text S2:** The methodological details for health risk assessment [1].

(1) Exposure assessment

The average daily intake (ADI) was calculated as recommended by USEPA:

$$ADI = \frac{C \times IR \times EF \times ED}{BW \times AT}$$

where C in this study represents the Cr concentration in soil (mg/kg), IR is the ingestion rate (kg/day), EF is the exposure frequency (day/year), ED is the exposure duration (year), BW is the body weight (kg), and AT is the time period over which the dose is averaged (day).

For heavy metals in contaminated soils, ingestion and dermal absorption are the most considered potential exposure pathways [2-4]. The exposure dose of the two pathways was calculated as follows:

Ingestion:

$$ADI_{ing} = \frac{C \times SIR \times EF \times ED}{BW \times AT}$$

where $ADI_{ing}$ is the average daily intake of Cr from soil ingestion (mg/kg-day) and SIR is the ingestion rate of soil (mg/day).

Dermal absorption:

$$ADI_{der} = \frac{C \times SA \times AF \times ABS \times EF \times ED}{BW \times AT}$$

where $ADI_{der}$ is the average daily intake of Cr from dermal absorption (mg/kg-day), SA is the exposed skin surface area ($cm^2$), AF is the adherence factor ($mg/cm^2$-day), and ABS is the dermal absorption factor (unitless).

(2) Non-carcinogenic risk assessment

Non-carcinogenic hazards are quantified by the hazard quotient (HQ). The HQ is calculated by comparing ADI with the reference dose (RfD). The specific calculation equation is:

$$HQ = \frac{ADI}{RfD}$$

where RfD is the chronic reference dose for Cr (mg/kg-day).

Due to lack of available reference doses for dermal absorption exposure, the USEPA has developed a method to extrapolate oral toxicity values for use in dermal risk assessment. $RfD_{ABS}$ is calculated as:

$$RfD_{ABS} = RfD_{O} \times ABS_{GI}$$

where $RfD_{ABS}$ is the adjusted reference dose for dermal absorption exposure (mg/kg-day), RfDo is the oral reference dose (mg/kg-day), and $ABS_{GI}$ is the gastro-intestinal absorption factor (unitless).

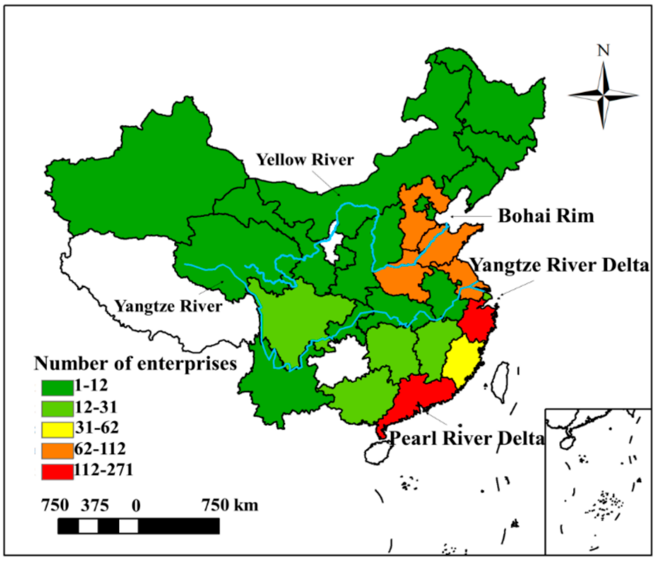


**Figure S1.** Distribution of major Cr-related industries in China.

(Data provided by Wang et al. [5])

**References**

1. United States Environmental Protection Agency (USEPA). *Risk Assessment Guidance for Superfund volume I. Human Health Evaluation Manual (Part A)*; Office of Emergency and Remedial Response: Washington, DC, USA, 1989.
2. Li, Z.; Ma, Z.; van der Kuijp, T.J.; Yuan, Z.; Huang, L. A Review of Soil Heavy Metal Pollution from Mines in China: Pollution and Health Risk Assessment. Sci. Total Environ. 2014, 468–469, 843–853. https://doi.org/10.1016/j.scitotenv.2013.08.090.
3. Yang, Q.; Li, Z.; Lu, X.; Duan, Q.; Huang, L.; Bi, J. A Review of Soil Heavy Metal Pollution from Industrial and Agricultural Regions in China: Pollution and Risk Assessment. Sci. Total Environ. 2018, 642, 690–700. https://doi.org/10.1016/j.scitotenv.2018.06.068.
4. Peng, J.; Zhang, S.; Han, Y.; Bate, B.; Ke, H.; Chen, Y. Soil Heavy Metal Pollution of Industrial Legacies in China and Health Risk Assessment. Sci. Total Environ. 2022, 816, 151632. https://doi.org/10.1016/j.scitotenv.2021.151632.
5. Wang, X.; Li, L.; Yan, X.; Tian, Y. Progress in Remediation of Chromium-contaminated Sites. Environ. Eng. 2020, 38, 1–8, 23. (In Chinese with English Abstract). https://doi.org/10.13205/j.hjgc.202006001.
